# No phylogenetic evidence for angiosperm mass extinction at the Cretaceous–Palaeogene (K-Pg) boundary

**DOI:** 10.1098/rsbl.2023.0314

**Published:** 2023-09-13

**Authors:** Jamie B. Thompson, Santiago Ramírez-Barahona

**Affiliations:** ^1^ The Milner Centre for Evolution, Department of Life Sciences, University of Bath, Bath, UK; ^2^ Instituto de Biología, Universidad Nacional Autónoma de México, Ciudad de México, Mexico

**Keywords:** mass extinction, angiosperms, K-Pg, diversification, extinction, macroevolution

## Abstract

The Cretaceous–Palaeogene mass extinction event (K-Pg) witnessed upwards of 75% of animal species going extinct, most notably among these are the non-avian dinosaurs. A major question in macroevolution is whether this extinction event influenced the rise of flowering plants (angiosperms). The fossil record suggests that the K-Pg event had a strong regional impact on angiosperms with up to 75% species extinctions, but only had a minor impact on the extinction rates of major lineages (families and orders). Phylogenetic evidence for angiosperm extinction dynamics through time remains unexplored. By analysing two angiosperm mega-phylogenies containing approximately 32 000–73 000 extant species, here we show relatively constant extinction rates throughout geological time and no evidence for a mass extinction at the K-Pg boundary. Despite high species-level extinction observed in the fossil record, our results support the macroevolutionary resilience of angiosperms to the K-Pg mass extinction event via survival of higher lineages.

## 1. Introduction

Mass extinctions are characterized by the massive loss of species diversity (75–90%) in a relatively short time span and exceedingly high rates (relative to background rates) of disappearance of higher taxonomic groups [1]. At least five major mass extinction events have punctuated the history of life and have profoundly shaped the diversity and distribution of entire groups of organisms. The most recent of these events was the Cretaceous–Palaeogene mass extinction (K-Pg) that occurred approximately 66 million years ago (Mya) and is associated with the Chicxulub Impact Event [2]. This event led to the demise of non-avian dinosaurs and high extinction rates of vertebrate species [1,3–6].

High-resolution fossil records suggest that, despite initial regional massive extinction of angiosperm species [7,8], most of their major extant lineages (i.e. orders, families) originated during the Cretaceous, survived the K-Pg event, and eventually recovered in diversity during the Palaeocene. The rise to ecological dominance of angiosperms accelerated after the K-Pg [8–10] and altered evolutionary trajectories of major lineages of plants, animals, and fungi [11–14]. Today, angiosperms dominate terrestrial biomes globally with a staggering approximately 290 000 species representing approximately 78% of all terrestrial plants [15].

The fossil record, despite being biassed throughout time and across space, provides a physical record of evolution [16]. But the angiosperm record is relatively sparse geographically and taxonomically, and confidently assigning taxonomic affinities is often difficult below the level of higher taxonomic groups [17,18]. This makes fossil-based macroevolutionary modelling difficult and not representative of angiosperm diversity as a whole. In analyses of macrofossils assigned to lower taxonomic levels, relatively low and stable extinction rates shape angiosperm history with no significant variation across the K-Pg boundary [18,19], in contrast to higher extinction rates in non-flowering vascular plants [18]. These global macroevolutionary trends contrast with high-resolution palaeobotanical records of regional angiosperm assemblages (micro and macrofossils), which often document sharp declines in abundance and elevated species-level extinctions at or shortly before the K-Pg, and high floristic turnover across the boundary [8,10,20–22]. These signatures tend to be concentrated in regions close to the Chicxulub impact zone [8,10], with further sites providing less or even no evidence of mass extinction at K-Pg [23]. Despite this, there is evidence of angiosperm species die-off in Patagonia and New Zealand [20,21,24]. Further support for angiosperm species extinctions at K-Pg are found in dynamics of unrelated lineages and geological processes: herbivorous insect diversity decreased following K-Pg [11], while abundance of ferns spores peaked [7]; denudation of terrestrial ecosystems across K-Pg [25] and occurrence of local erosive events [26].

Assessing the impacts of the K-Pg event on angiosperm extinction at higher taxonomic levels is difficult because of geographical bias and poor taxonomic resolution in the fossil record [8], but the available evidence indicates a rapid recovery of nearly all Cretaceous higher angiosperm taxa after the K-Pg [7,8,21,22]. Assessing macroevolutionary dynamics of angiosperms using phylogenetic evidence could provide key insights into the impacts of K-Pg and how this event triggered the restructuring of all terrestrial biomes and the emergence of modern-day ecosystems [10,27]. These questions remain poorly explored and the impact of the K-Pg on major angiosperm lineages remains one of the major unanswered questions in angiosperm macroevolution [28].

**Figure 1.**
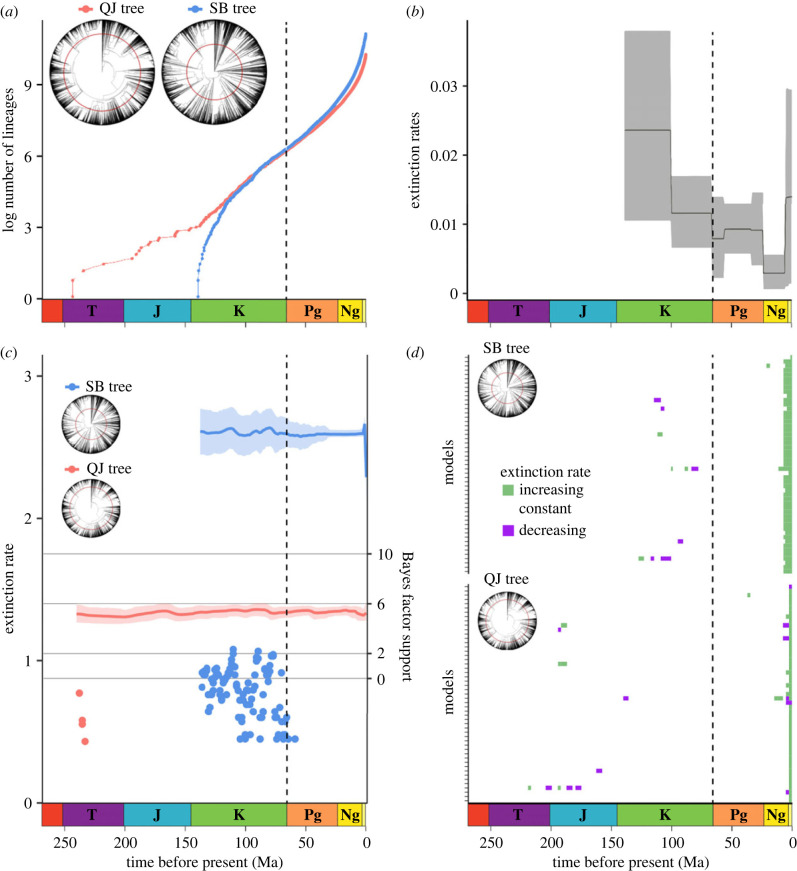
Macroevolutionary dynamics of angiosperms not negatively impacted at the K-Pg mass extinction event. Lineage through time plots (*a*) and phylogenies comprising approximately 32 000 and approximately 73 000 species (denoted as QJ and SB, respectively) indicate no apparent change across K-Pg. Generic-level extinction rates of angiosperms estimated with PyRate (*b*), adapted from [18], suggesting no significant difference in extinction rate following K-Pg. Phylogenetic extinction rates estimated by CoMET showing the detected mass extinctions with low support (Bayes factor lower than 6) (*c*), demonstrating that phylogenies do not support a mass extinction at the K-Pg. Trends in alternative models within congruence classes of both trees (*d*), showing that a pattern of resilience to K-Pg is supported despite non-identifiability of diversification rates. The geological timescale is visualized in each panel, and K-Pg is represented by vertical dashed lines in the plots and concentric lines in the phylogenies.

Here, we applied the Bayesian method CoMET [29] to assess the influence of the K-Pg event on angiosperm extinction rates. To capture some of the phylogenetic and age uncertainties, we analysed the two largest angiosperm-wide mega-phylogenies, both of which sample a substantial portion of angiosperm diversity: approximately 32 000 (approx. 10.5%) and approximately 70 000 (approx. 25.2%) species, respectively [30–32]. Backbone topology and estimated divergence times differ among these trees (figure 1), and the age of crown group angiosperms (approx. 243.3 and approx. 139.4 Mya, respectively) spans the plausible interval of 140–270 Ma proposed for the group [33].

## 2. Results and discussion

Our Bayesian analyses of diversification through time provide congruent results between two very different mega-phylogenies and strengthen the evidence, as revealed by fossils [7,8], of the limited impact of the K-Pg on the extinction of major angiosperm lineages. The analyses revealed relatively stable extinction rates through time and no evidence of mass extinctions (figure 1). Overwhelming evidence (Bayes factor, BF) supported models with stable diversification rates over models incorporating mass extinctions (BF_SB_ > 65 000; BF_QJ_ > 268 000). This agrees with the most recent Bayesian analyses of the macrofossil record that suggests a relatively low impact of the K-Pg mass extinction on angiosperm diversity at higher taxonomic levels [18].

The analyses we employed have successfully detected signatures of mass extinctions in large phylogenies of extant species [29,34,35], gaining macroevolutionary insights in the face of substantial taphonomic biases (lineages and regions with a poor fossil record; e.g. [36]). However, phylogenies are reconstructions, not observations, and suffer from multiple sources of uncertainty [37] that represent challenges when exploring deep-time dynamics. For instance, different time-calibrated phylogenies for mammals support contrasting models for extinction rates across the K-Pg boundary, supporting either stable diversification rates across the K-Pg [38] or macroevolutionary trends being shaped by the K-Pg mass extinction [39]. The vast majority of angiosperm lineages have a poor fossil record, therefore extant phylogenies are the only source of data to conduct analyses on their macroevolutionary dynamics, yet inferences drawn from such phylogenetic data are sensitive to numerous sources of uncertainty. These uncertainties are magnified in angiosperms due to their extreme richness, complex evolution, and unresolved backbone topology and the timings of early divergences [28,33]. Analysing a sample of plausible angiosperm-wide phylogenies is currently intractable, but the two mega-phylogenies we used capture wide variation in divergence estimates while comprehensively sampling extant diversity and covering the deeper nodes in the phylogeny, which is crucial for the hypotheses we tested: the SB tree samples greater than 10 000 of the known greater than 13 000 genera in 401 of 416 families [40], while QJ samples approximately 7900 genera in 401 families. Regardless of which crown group age estimate for angiosperms we considered, numerous major lineages survive the K-Pg [8,27], and no difference in dynamics were recovered between phylogenies (figure 1).

Our results support the resilience of major angiosperm lineages to the K-Pg extinction event, contrasting with phylogenetic evidence for other lineages such as non-avian dinosaurs, conifers [29] and fishes [34], all of which appear to have been impacted by the K-Pg event at higher taxonomic levels. The resilience of major angiosperm lineages to the K-Pg event is likely associated with the acquisition of eco-morphological innovations (e.g. CAM photosynthesis, wind and insect pollination, animal dispersal) and ecological opportunity following the K-Pg [41–45]. We suggest that the wide range of ecological niches occupied by angiosperms since the Late Cretaceous [14,28] likely promoted lineage diversification before the K-Pg and favoured their eventual survival when confronted with major environmental changes triggered by the K-Pg.

Analyses of diversification in deep time suffer from non-identifiability [46], in which infinite combinations of speciation and extinction rates can fit a phylogeny with the same likelihood scores (congruent classes). To assess the impact of non-identifiability we tested whether constant extinction rates are supported among alternative models within the same congruence class [47]. We found no evidence of rate variation across K-Pg within congruent classes, supporting constant extinction trends (figure 1). It is important to note that CoMET models tree-wide diversification rates; previous studies have evidenced a high degree of heterogeneity in diversification rates (both accelerations and decelerations) across the angiosperm tree [48,49]. Several diversification rate shifts in angiosperms appear to temporarily overlap the K-Pg. Applying the methods used here to particular angiosperm lineages could reveal variation in macroevolutionary dynamics associated with geographical ranges and eco-morphological traits, such as high-latitudinal distributions [50], insect pollination [43] and polyploidy [51]. Whether distinct clades within angiosperms were differentially impacted by the K-Pg mass extinction remains an open question, particularly regarding differences among lineages characterized by particular geographical ranges and eco-morphological traits.

How do we reconcile the apparent macroevolutionary stability of angiosperms with the fossil evidence showing high levels of species extinction following the K-Pg event? It is clear from the palaeobotanical record, that the K-Pg extinction event caused widespread plant species-level extinctions and changes in ecosystem composition at local and regional scales, such as tropical rainforests [10,22]. The angiosperm fossil record reveals a high rate of species turnover (which entails elevated species-level extinctions) across the K-Pg, but without loss of Cretaceous higher-taxon diversity [7,8]. While angiosperms as a whole appeared to have had stable macroevolutionary dynamics across the K-Pg, it is important to understand the heterogeneous impacts this event may have had in different angiosperm lineages and the taxonomic scales involved.

While species-level dynamics of entire fossil floras can be thoroughly studied, most of these floras remain poorly known taxonomically due to difficulties in assigning specimens to higher taxonomic levels [17]. On the contrary, in phylogenetics, by necessity and as a function of how deep we look into the past, we can only search for signatures of extinction at higher taxonomic ranks (lineages). Our results partially agree with the inferences that most extant family-level diversity predates the K-Pg event [27] and with palaeobotanical evidence placing fossil representatives of extant families both before and after the K-Pg, but with high rates of species turnover [7,8,10]. There are possible signatures of elevated rates of turnover in the shape of the angiosperm backbone phylogeny. The protracted phylogenetic fuses (the time elapsed between the stem and crown node) inferred for extant angiosperm families, most of which predate the K-Pg [27], are consistent with models with high extinction rates coupled to elevated turnover ([52]; but see [53]). However, this remains a still unresolved question in angiosperm phylogenetics.

One of the main problems when assessing mass extinction episodes is the definition of what these events entail, and how we measure them. Generally, mass extinctions are characterized and measured by high loss of species diversity that often follow the disappearance of entire taxonomic groups from the palaeontological record. As evidenced by angiosperms, high extinction rates at the species level can be disassociated from the loss of entire taxonomic groups. The fossil record indicates that the K-Pg led to mass extinction at the species level, but with no taxonomic selectivity leading to the disappearance of major angiosperm lineages [7,8,10,22]. Although we support the survival of higher lineages across K-Pg, our results do not reject species-level extinctions. Here we argue that the apparent contradiction between palaeobotanical and phylogenetic evidence of extinctions emerges, at least partially, from the interchange between these two taxonomic dimensions of mass extinctions. This highlights the importance of integrating both phylogenetic and fossil evidence to complement our understanding of angiosperm macroevolution and their rise to dominance across the K-Pg.

## 3. Material and methods

### (a) Diversification analyses

We used updated versions of the molecular mega-phylogenies produced by Zanne *et al*. [30] and Smith & Brown [32]; non-angiosperm species were pruned prior to analyses. Qian & Jin [31] corrected the taxonomy of the Zanne *et al*. [30] mega phylogeny, removed duplicates and added six families to extend coverage to all currently recognized families. Igea & Tanentzap [54] standardized the taxonomy of the Smith & Brown [32] mega-phylogeny against ‘The Plant List V1.1' [55]. Further details on molecular and taxonomic sampling in both trees are available in the electronic supplementary material.

We produced log-transformed lineage through time (LTT) plots for both mega-phylogenies using the *phytools* package [56] in R [57]. We compared support for models of constant diversification and mass extinction at approximately 66 Ma in both phylogenies with marginal likelihoods, estimated with stepping-stone sampling in the TESS package [58]. In each model we specified the fraction of sampled species, ran 1000 iterations with burn-in of 100, implemented 50 stepping-stones and estimated Bayes factor support between models.

We estimated diversification dynamics with mass extinctions using the CoMET model [29] implemented in TESS [58]. A threshold of instantaneous species-loss of 75% with a beta-distribution for survival probability spanning approximately 18 to approximately 32% was implemented using a compound Poisson process; a loss of 75% diversity is a relatively relaxed threshold but agrees with estimates of species-loss during the K-Pg event. Incomplete sampling was accounted for by specifying the fraction of sampled species, the number of expected rate changes was set to 25 (slightly more conservative than [49]), the number of expected mass extinctions was set to one, and empirical hyperpriors for speciation and extinction rates were estimated automatically with an initial MCMC run. The final analyses were run in replicate to convergence until an effective sample size greater than 300 was achieved; the first 10 000 generations of each run were discarded as burn-in. Further details on model selection and parameter choices are available in the electronic supplementary material.

### (b) Sensitivity testing

Sensitivity testing was performed in CoMET using the smaller phylogeny [30,31] by replicating analyses and altering the number of expected rate changes five times from 1 to 1000 (1, 100, 200, 500, 1000). Further analyses were undertaken to account for the non-identifiability of diversification rates [46] with the CRABS [47] package in R. Congruence classes of the CoMET parameters were explored with 500 models, assuming a constant rate of extinction, and in the absence of any linear or exponential temporal trend. We sampled and visualized 50 models for each mega-phylogeny to reduce crowding in the figure.

## Data Availability

Data are available from the Dryad Digital Repository: http://dx.doi.org/10.5061/dryad.n8pk0p329 [59]. The data are provided in the electronic supplementary material [60].

## References

[1] Raup DM, Sepkoski JJ. 1982 Mass extinctions in the marine fossil record. Science **215**, 1501-1503. (10.1126/science.215.4539.1501) 17788674

[2] Schulte P et al. 2010 The Chicxulub asteroid impact and mass extinction at the Cretaceous–Paleogene boundary. Science **327**, 1214-1218. (10.1126/science.1177265) 20203042

[3] Longrich NR, Tokaryk T, Field DJ. 2011 Mass extinction of birds at the Cretaceous–Paleogene (K–Pg) boundary. Proc. Natl Acad. Sci. USA **108**, 15 253-15 257. (10.1073/pnas.1110395108) PMC3174646 21914849

[4] Longrich NR, Bhullar B-AS, Gauthier JA. 2012 Mass extinction of lizards and snakes at the Cretaceous–Paleogene boundary. Proc. Natl Acad. Sci. USA **109**, 21 396-21 401. (10.1073/pnas.1211526110) PMC3535637 23236177

[5] Longrich NR, Scriberas J, Wills MA. 2016 Severe extinction and rapid recovery of mammals across the Cretaceous–Palaeogene boundary, and the effects of rarity on patterns of extinction and recovery. J. Evol. Biol. **29**, 1495-1512. (10.1111/jeb.12882) 27167897

[6] Lyson TR et al. 2019 Exceptional continental record of biotic recovery after the Cretaceous–Paleogene mass extinction. Science **366**, 977-983. (10.1126/science.aay2268) 31649141

[7] Nichols DJ, Johnson KR. 2008 Plants and the K-T boundary. Cambridge, UK: Cambridge University Press.

[8] Wilf P, Carvalho MR, Stiles E. 2023 The end-Cretaceous plant extinction: heterogeneity, ecosystem transformation, and insights for the future. Cambridge Prisms: Extinction **1**, e14. (10.1017/ext.2023.13) PMC11895728 40078678

[9] Krug AZ, Jablonski D, Valentine JW. 2009 Signature of the end-Cretaceous mass extinction in the modern biota. Science **323**, 767-771. (10.1126/science.1164905) 19197060

[10] Carvalho MR et al. 2021 Extinction at the end-Cretaceous and the origin of modern Neotropical rainforests. Science **372**, 63-68. (10.1126/science.abf1969) 33795451

[11] Labandeira CC, Johnson KR, Wilf P. 2002 Impact of the terminal Cretaceous event on plant–insect associations. Proc. Natl Acad. Sci. USA **99**, 2061-2066. (10.1073/pnas.042492999) 11854501 PMC122319

[12] Barba-Montoya J, dos Reis M, Schneider H, Donoghue PC, Yang Z. 2018 Constraining uncertainty in the timescale of angiosperm evolution and the veracity of a Cretaceous terrestrial revolution. New Phytol. **218**, 819-834. (10.1111/nph.15011) 29399804 PMC6055841

[13] Condamine FL, Silvestro D, Koppelhus EB, Antonelli A. 2020 The rise of angiosperms pushed conifers to decline during global cooling. Proc. Natl Acad. Sci. USA **117**, 28 867-28 875. (10.1073/pnas.2005571117) PMC7682372 33139543

[14] Benton MJ, Wilf P, Sauquet H. 2021 The Angiosperm Terrestrial Revolution and the origins of modern biodiversity. New Phytol. **233**, 2017-2035. (10.1111/nph.17822) 34699613

[15] Christenhusz MJM, Byng JW. 2016 The number of known plants species in the world and its annual increase. Phytotaxa **261**, 201. (10.11646/phytotaxa.261.3.1)

[16] Xing Y et al. 2016 Testing the biases in the rich Cenozoic angiosperm macrofossil record. Int. J. Plant Sci. **177**, 371-388. (10.1086/685388)

[17] Wilf P. 2008 Fossil angiosperm leaves: paleobotany's difficult children prove themselves. Paleontol. Soc. Papers **14**, 319-333. (10.1017/s1089332600001741)

[18] Silvestro D, Cascales-Miñana B, Bacon CD, Antonelli A. 2015 Revisiting the origin and diversification of vascular plants through a comprehensive Bayesian analysis of the fossil record. New Phytol. **207**, 425-436. (10.1111/nph.13247) 25619401 PMC4949670

[19] Crepet WL, Niklas KJ. 2009 Darwin's second ‘abominable mystery’: why are there so many angiosperm species? Amer. J. Bot. **96**, 366-381. (10.3732/ajb.0800126) 21628194

[20] Johnson KR. 1992 Leaf-fossil evidence for extensive floral extinction at the Cretaceous–Tertiary boundary, North Dakota, USA. Cretaceous Res. **13**, 91-117. (10.1016/0195-6671(92)90029-p)

[21] Vajda V, Raine JI, Hollis CJ. 2001 Indication of global deforestation at the Cretaceous–Tertiary boundary by New Zealand fern spike. Science **294**, 1700-1702. (10.1126/science.1064706) 11721051

[22] Stiles E, Wilf P, Iglesias A, Gandolfo MA, Cúneo NR. 2020 Cretaceous–Paleogene plant extinction and recovery in Patagonia. Paleobiology **46**, 445-469. (10.1017/pab.2020.45)

[23] De Benedetti F, Zamaloa MC, Gandolfo MA, Cúneo NR. 2023 Pollen from the K–Pg boundary of the La Colonia Formation, Patagonia, Argentina. Rev. Palaeobot. Palynol. **316**, 104933. (10.1016/j.revpalbo.2023.104933)

[24] Vajda V, McLoughlin S. 2004 Fungal proliferation at the Cretaceous–Tertiary boundary. Science **303**, 1489-1489. (10.1126/science.1093807) 15001770

[25] Misra S, Froelich PN. 2012 Lithium isotope history of Cenozoic seawater: changes in silicate weathering and reverse weathering. Science **335**, 818-823. (10.1126/science.1214697) 22282473

[26] Fastovsky DE, Dott Jr RH. 1986 Sedimentology, stratigraphy, and extinctions during the Cretaceous–Paleogene transition at Bug Creek, Montana. Geology **14**, 279. (10.1130/0091-7613(1986)14<279:ssaedt>2.0.co;2)

[27] Ramírez-Barahona S, Sauquet H, Magallón S. 2020 The delayed and geographically heterogeneous diversification of flowering plant families. Nat. Ecol. Evol. **4**, 1232-1238. (10.1038/s41559-020-1241-3) 32632260

[28] Sauquet H, Magallón S. 2018 Key questions and challenges in angiosperm macroevolution. New Phytol. **219**, 1170-1187. (10.1111/nph.15104) 29577323

[29] May MR, Höhna S, Moore BR. 2015 A Bayesian approach for detecting mass-extinction events when rates of lineage diversification vary. Methods Ecol. Evol. **7**, 947-959. (10.1101/020149)

[30] Zanne AE et al. 2013 Three keys to the radiation of angiosperms into freezing environments. Nature **506**, 89-92. (10.1038/nature12872) 24362564

[31] Qian H, Jin Y. 2015 An updated megaphylogeny of plants, a tool for generating plant phylogenies and an analysis of phylogenetic community structure. J. Plant Ecol. **9**, 233-239. (10.1093/jpe/rtv047)

[32] Smith SA, Brown JW. 2018 Constructing a broadly inclusive seed plant phylogeny. Amer. J. Bot. **105**, 302-314. (10.1002/ajb2.1019) 29746720

[33] Sauquet H, Ramírez-Barahona S, Magallón S. 2022 What is the age of flowering plants? J. Exp. Bot. **73**, 3840-3853. (10.1093/jxb/erac130) 35438718

[34] Arcila D, Tyler JC. 2017 Mass extinction in tetraodontiform fishes linked to the Palaeocene–Eocene Thermal Maximum. Proc. R. Soc. B **284**, 20171771. (10.1098/rspb.2017.1771) PMC5698648 29118135

[35] Kopperud BT, Magee AF, Höhna S. 2023 Rapidly changing speciation and extinction rates can be inferred in spite of nonidentifiability. Proc. Natl Acad. Sci. USA **120**, e2208851120. (10.1073/pnas.2208851120) 36757894 PMC9963352

[36] Hernández-Hernández T, Brown JW, Schlumpberger BO, Eguiarte LE, Magallón S. 2014 Beyond aridification: multiple explanations for the elevated diversification of cacti in the new world succulent biome. New Phytol. **202**, 1382-1397. (10.1111/nph.12752) 24611540

[37] Rangel TF, Colwell RK, Graves GR, Fučíková K, Rahbek C, Diniz-Filho JA. 2015 Phylogenetic uncertainty revisited: implications for ecological analyses. Evolution **69**, 1301-1312. (10.1111/evo.12644) 25800868

[38] Bininda-Emonds OR et al. 2007 The delayed rise of present-day mammals. Nature **446**, 507-512. (10.1038/nature05634) 17392779

[39] Meredith RW et al. 2011 Impacts of the Cretaceous terrestrial revolution and KPG extinction on mammal diversification. Science **334**, 521-524. (10.1126/science.1211028) 21940861

[40] Byng JW et al. 2016 An update of the Angiosperm Phylogeny Group classification for the orders and families of flowering plants: APG IV. Bot. J. Linn. Soc. **181**, 1-20. (10.1111/boj.12385)

[41] Eriksson O, Friis EM, Löfgren P. 2000 Seed size, fruit size, and dispersal systems in angiosperms from the Early Cretaceous to the Late Tertiary. Am. Nat. **156**, 47-58. (10.1086/303367) 10824020

[42] Friis EM, Pedersen KR, Crane PR. 2006 Cretaceous angiosperm flowers: innovation and evolution in plant reproduction. Palaeogeogr. Palaeoclimatol. Palaeoecol. **232**, 251-293. (10.1016/j.palaeo.2005.07.006)

[43] McElwain JC, Punyasena SW. 2007 Mass extinction events and the plant fossil record. Trends Ecol. Evol. **22**, 548-557. (10.1016/j.tree.2007.09.003) 17919771

[44] Soltis PS, Folk RA, Soltis DE. 2019 Darwin review: angiosperm phylogeny and evolutionary radiations. Proc. R. Soc. B **286**, 20190099. (10.1098/rspb.2019.0099)

[45] Thompson JB, Davis KE, Dodd HO, Wills MA, Priest NK. 2023 Speciation across the Earth driven by global cooling in terrestrial orchids. Proc. Natl Acad. Sci. USA **120**, e2102408120. (10.1073/pnas.2102408120) 37428929 PMC10629580

[46] Louca S, Pennell MW. 2020 Extant timetrees are consistent with a myriad of diversification histories. Nature **580**, 502-505. (10.1038/s41586-020-2176-1) 32322065

[47] Höhna S, Kopperud BT, Magee AF. 2022 CRABS: congruent rate analyses in birth–death scenarios. Methods Ecol. Evol. **13**, 2709-2718. (10.1111/2041-210x.13997)

[48] Tank DC, Eastman JM, Pennell MW, Soltis PS, Soltis DE, Hinchliff CE, Brown JW, Sessa EB, Harmon LJ. 2015 Nested radiations and the pulse of angiosperm diversification: Increased diversification rates often follow whole genome duplications. New Phytol. **207**, 454-467. (10.1111/nph.13491) 26053261

[49] Magallón S, Sánchez-Reyes LL, Gómez-Acevedo SL. 2018 Thirty clues to the exceptional diversification of flowering plants. Ann. Bot. **123**, 491-503. (10.1093/aob/mcy182) PMC6377106 30376040

[50] McLoughlin S, Carpenter RJ, Jordan GJ, Hill RS. 2008 Seed ferns survived the end-Cretaceous mass extinction in Tasmania. Amer. J. Bot. **95**, 465-471. (10.3732/ajb.95.4.465) 21632371

[51] Fawcett JA, Maere S, Van de Peer Y. 2009 Plants with double genomes might have had a better chance to survive the Cretaceous–Tertiary extinction event. Proc. Natl Acad. Sci. USA **106**, 5737-5742. (10.1073/pnas.0900906106) 19325131 PMC2667025

[52] Budd GE, Mann RP. 2018 History is written by the victors: the effect of the push of the past on the fossil record. Evolution **72**, 2276-2291. (10.1111/evo.13593) 30257040 PMC6282550

[53] Beavan AJ, Pisani D, Donoghue PC. 2021 Diversification dynamics of total-, stem-, and crown-groups are compatible with molecular clock estimates of Divergence Times. Sci. Adv. **7**, eabf2257. (10.1126/sciadv.abf2257) 34117058 PMC8195484

[54] Igea J, Tanentzap AJ. 2020 Angiosperm speciation cools down in the Tropics. Ecol. Lett. **23**, 692-700. (10.1111/ele.13476) 32043734 PMC7078993

[55] Jin Y, Qian H. 2019V PhyloMaker: an R package that can generate very large phylogenies for vascular plants. Ecography **42**, 1353-1359. (10.1111/ecog.04434) PMC9363651 35967255

[56] Revell LJ. 2011 Phytools: an R package for phylogenetic comparative biology (and other things). Methods Ecol. Evol. **3**, 217-223. (10.1111/j.2041-210x.2011.00169.x)

[57] R Core Team. 2022 R: a language and environment for statistical computing. Vienna, Austria: R Foundation for Statistical Computing. (https://www.R-project.org/).

[58] Höhna S, May MR, Moore BR. 2015 TESS: An R package for efficiently simulating phylogenetic trees and performing Bayesian inference of lineage diversification rates. Bioinformatics **32**, 789-791. (10.1093/bioinformatics/btv651) 26543171

[59] Thompson JB, Ramírez-Barahona S. 2023 Data from: No phylogenetic evidence for angiosperm mass extinction at the Cretaceous–Palaeogene (K-Pg) boundary. Dryad Digital Repository. (10.5061/dryad.n8pk0p329) PMC10498348 37700701

[60] Thompson JB, Ramírez-Barahona S. 2023 No phylogenetic evidence for angiosperm mass extinction at the Cretaceous–Palaeogene (K-Pg) boundary. Figshare. (10.6084/m9.figshare.c.6806622)

